# Membrane Technologies for Lactic Acid Separation from Fermentation Broths Derived from Renewable Resources

**DOI:** 10.3390/membranes8040094

**Published:** 2018-10-12

**Authors:** Maria Alexandri, Roland Schneider, Joachim Venus

**Affiliations:** Leibniz Institute for Agricultural Engineering and Bioeconomy (ATB), Max-Eyth-Allee 100, 14469 Potsdam, Germany; malexandri@atb-potsdam.de (M.A.); rschneider@atb-potsdam.de (R.S.)

**Keywords:** lactic acid, microfiltration, nanofiltration, electrodialysis, purification

## Abstract

Lactic acid (LA) was produced on a pilot scale using a defined medium with glucose, acid whey, sugar bread and crust bread. The fermentation broths were then subjected to micro- and nanofiltration. Microfiltration efficiently separated the microbial cells. The highest average permeate flow flux was achieved for the defined medium (263.3 L/m^2^/h) and the lowest for the crust bread-based medium (103.8 L/m^2^/h). No LA losses were observed during microfiltration of the acid whey, whilst the highest retention of LA was 21.5% for crust bread. Nanofiltration led to high rejections of residual sugars, proteins and ions (sulphate, magnesium, calcium), with a low retention of LA. Unconverted sugar rejections were 100% and 63% for crust bread and sugar bread media respectively, with corresponding LA losses of 22.4% and 2.5%. The membrane retained more than 50% of the ions and proteins present in all media and more than 60% of phosphorus. The average flux was highly affected by the nature of the medium as well as by the final concentration of LA and sugars. The results of this study indicate that micro- and nanofiltration could be industrially employed as primary separation steps for the biotechnologically produced LA.

## 1. Introduction

Bio-based production of platform chemicals from renewable resources is currently a topic of intensive research. Lactic acid is an important bulk chemical with a global market of 1220.0 kt in 2016 [1]. The lactic acid market presents an annual growth of 16.2%, mainly due to the production of polylactic acid and ethyl lactate, whilst the estimated demand for 2025 is 1960.1 kt [1,2]. Lactic acid can be produced biotechnologically from various renewable resources and waste streams such as food waste [3,4,5], mixed bakery waste [6], coffee pulp and mucilage [7,8], algal biomass [9], and lignocellulosic hydrolysates [10] among others.

The utilization of waste and by-product streams as alternative fermentation substrates could reduce the cost of the upstream process of bulk chemicals and, at the same time, their production would not compete with food and feed. On the other hand, renewable materials like lignocellulosic biomass result in an undefined medium, rendering the downstream process of organic acids more complicated. For lactic acid, the cost for its separation and purification from the fermentation broth can reach 50% of the total process costs [1]. Calcium precipitation was the most common method for organic acid separation but large quantities of gypsum are generated and the resultant lactic acid presents low purity [1]. Various separation methods have been proposed in the literature so far, including solvent extraction, membrane separation, ion exchange chromatography, and reactive distillation aiming to overcome these drawbacks [1].

Membrane separation techniques have recently attracted attention as they are environmentally benign and easily scaled up [11]. Microfiltration (MF) is an effective method for the removal of microbial biomass, while nanofiltration (NF) efficiently retains proteins and other macromolecules as well as multivalent anions [11,12]. Many researchers have already demonstrated lactic acid separation using NF techniques either from fermentation broths or from defined solutions [13].

The aim of this study is to demonstrate the efficient separation of lactic acid from different fermentation broths. In all cases, lactic acid fermentation was carried out on a pilot scale using three inexpensive substrates (crust bread, sugar bread, and acid whey) or a defined glucose solution. MF and NF were applied in all cases aiming to separate lactic acid from other fermentation components.

## 2. Materials and Methods

### 2.1. Microorganisms

Different *Bacillus coagulans* strains were used for the fermentation of each medium according to the special substrate requirements. Strain A20 was isolated from potato washing water and tested for the fermentation of the defined medium. Strains A369 and A107 were isolated from rapeseed meal and were utilized for the fermentation of acid whey and sugar bread respectively. Finally, fermentation of crust bread was carried out by the strain A59, isolated from rye grain. An inoculum preparation was carried out in De Man, Rogosa and Sharpe (MRS) broth (Merck, Darmstadt, Germany) and 0.67 g Everzit Dol (Evers e.K., Hopsten, Germany) dolomite as a buffer. The strains were cultivated at 52 °C for 10–16 h, at 100 rpm on an orbital shaker.

### 2.2. Substrate Preparation

Sugar bread and crust bread were kindly provided by CETECE (Palencia, Spain). Starch hydrolysis was carried out using the commercial enzymatic preparations BAN 240L (Novozymes, Bagsvaerd, Denmark), Stargen 002 (Genencor, New York, NY, USA), Viscoferm (Novozymes, Bagsvaerd, Denmark), whilst Protease N-01 (ASA-Spezialenzyme GmbH, Wolfenbüttel, Germany) was added for protein hydrolysis. Acid whey was supplied by Glanbia Ingredients (Kilkenny, Ireland) and was readily utilized for fermentation.

### 2.3. Pilot Scale Fermentations

A pilot scale bioreactor with 1000 L capacity (A&B Heldrungen GmbH, Heldrungen, Germany) was employed for all of the fermentations, with different working volumes depending on the media applied. All fermentations were carried out in batch mode, at 52 °C, and the pH was adjusted to 6.0 using 20% NaOH (*w*/*w*). At the end of the fermentation, the medium was heated up to 85 °C for 1.5 h in order to deactivate the biomass. Inactivation was carried out using steam directly in the medium, resulting in an increase in the final volume.

Batch fermentations were carried out using four different substrates: a defined medium, acid whey, a sugar bread hydrolysate and a crust bread hydrolysate. For the preparation of the defined medium, 120 g/L of glucose was utilized as a carbon source with 15 g/L of yeast extract. The working volume of the fermentation was 700 L. Fermentation with acid whey was carried out using a 300 L working volume, in the presence of 10 g/L yeast extract. Sugar bread and crust bread hydrolysates were utilized as carbon and nutrient sources supplemented with 10 g/L and 5 g/L yeast extract, respectively. The working volume for both fermentations was 600 L. Inocula for all fermentations were 6% and the preparation was carried out under the same culture conditions in a 72 L BIOSTAT UD bioreactor (B-Braun Biotech, Melsungen, Germany). Samples were taken at regular intervals for the analysis of sugars and lactic acid. Inactivation of the samples was carried out in a water bath at 95 °C for 20 min.

### 2.4. Membranes and Experimental Procedures

**Figure 1:**
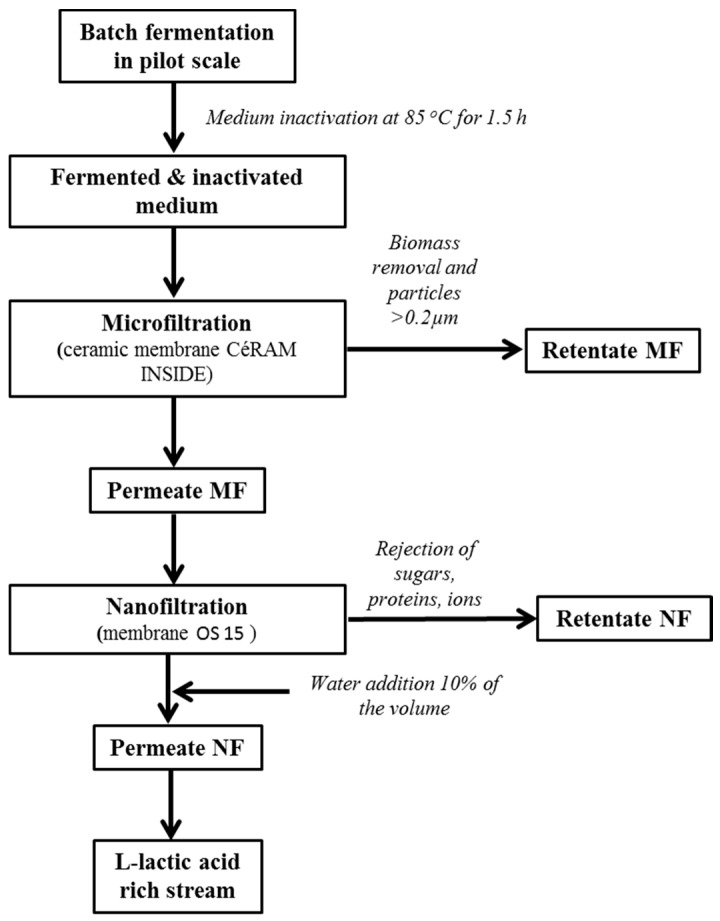
Schematic diagram of the studied process.

A schematic diagram describing the overall studied process is presented in Figure 1. After the inactivation of the biomass, the medium was filtrated using a UFI-TEC cross-flow microfiltration system (UFI-TEC GmbH, Oranienburg, Germany), operating at 1.5 bar and equipped with 4 ceramic membranes CéRAM INSIDE (TAMI Industries, Nyons, France), ZrO_2_-TiO_2_, with a pore size of 0.2 μm and a 6.65 m^2^ surface area. Losses of the medium during microfiltration were monitored (Table 1) due to some stops during the process.

The permeate stream obtained from the microfiltration was subjected to nanofiltration at 30 bar and at 30 °C using a UFI-TEC cross-flow nanofiltration system (UFI-TEC, GmbH, Oranienburg, Germany). The membrane utilized was OS 15 (DOW Chemical Company, Schwalbach, Germany) with a cut-off of 150–300 Da. According to the manufacturer, the membrane area was 1.7 m^2^ and the average permeate flow was 1.7 m^3^/day. This membrane presented a 96% salt rejection (using MgSO_4_), with a maximum working pressure of 4.14 MPa (when operating at temperatures <35 °C) and a 50 °C maximum working temperature. Water flux analysis was monitored at 6, 10 and 20 bar. The MF permeate was divided into batches of approximately 100 mL and then introduced to the nanofiltration membranes. The treated volumes are presented in Table 1. For every substrate, water was added close to the end of the filtration, aiming to reduce the viscosity of the retentate and decrease the loss of lactic acid. The amount of the water added was approximately 10% of the initial volume (Table 1).

### 2.5. Calculations

The permeate flux (J) for MF and NF was calculated using the following equation:

J = Q_p_/Am (1)

where Q_p_ is the filtrate flow rate through the membrane and Am is the surface area of the membrane. The permeate flux is expressed in L/h/m^2^.

The rejection percentage for a specified compound (sugars, lactic acid, proteins, ions) was calculated as follows:

R = 1 − C_p_/C (2)

where C_p_ is the concentration of the specified compound in the permeate stream and C is the corresponding concentration in the feed.

### 2.6. Analytical Methods

The concentration of lactic acid and sugars was analyzed by HPLC (DIONEX, Sunnyvale, CA, USA), using a Eurokat H column (300 mm × 8 mm × 10µm, Knauer, Berlin, Germany), with 5 mM H_2_SO_4_ as the mobile phase at a flow rate of 0.8 mL/min. The injection volume was 10 µL and detection was carried out using a refractive index detector (RI-71. Shodex/Shoko Science Co., Tokyo, Japan). The analysis of cations and anions was carried out by ion chromatography (DIONEX, Sunnyvale, CA, USA). Cation determination was achieved using an IonPac CS 16 column (250 mm × 4 µm, DIONEX, Sunnyvale, CA, USA), using 30 mM CH_3_SO_3_H at a flow rate of 1.0 mL/min, at 40 °C. Determination of anions was carried out with an IonPac AS9-HC column (250 mm × 4 µm, DIONEX, Sunnyvale, CA, USA), using 9 mM Na_2_CO_3_ as the mobile phase, at a flow rate of 1.2 mL/min, operating at room temperature.

The protein content of the samples was expressed as Kjeldahl-nitrogen and determination was carried out according to the DIN-EN-25663 standard method.

The total phosphorus (P) content was measured by flow injection analysis (FIA), according to the international standard ISO 15681-1, 2003.

## 3. Results and Discussion

### 3.1. Fermentation

**Figure 2:**
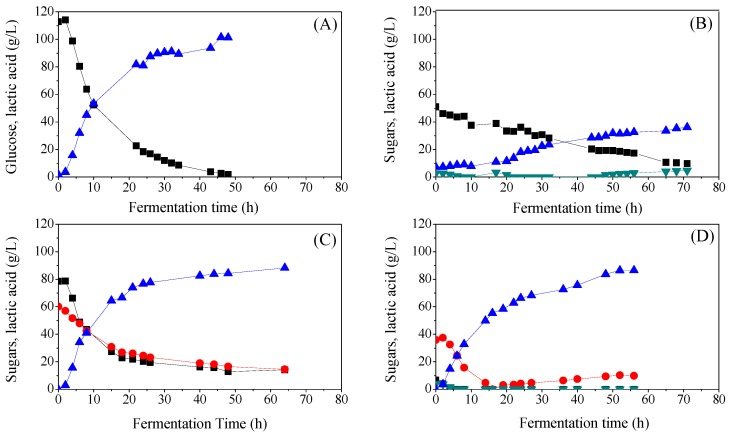
Sugar consumption and lactic acid production during fermentation of (**A**) defined medium; (**B**) acid whey; (**C**) sugar bread and (**D**) crust bread. Glucose and lactose (black square), galactose and fructose (dark cyan down-pointing triangle), sucrose and maltose (red cycle), lactic acid (blue up-pointing triangle).

The replacement of commercial sugars and nutrients with waste and by-product streams could not only decrease the upstream cost of the biotechnological production of lactic acid but it would also contribute to the sustainable production of platform chemicals in general. Figure 2 presents the kinetics of four individual batch fermentations in pilot scale, using (A) a defined medium with glucose, (B) acid whey, (C) sugar bread, and (D) crust bread. When a defined medium with glucose as the single carbon source was utilized, fermentation lasted for 48 h, resulting in 99.5 g/L of lactic acid with the strain A20 (Figure 2A). The lactic acid yield from the total glucose concentration was 0.87 g/g and the productivity was 2.11 g/L/h. For the fermentation using acid whey as a substrate, isolate A369 was selected since it has the ability to consume lactose. This medium also contained 7.3 g/L of lactic acid. Lactose consumption started during the first 2 h of fermentation but with a lower rate in comparison to glucose (Figure 2B). Some galactose was also detected in the broth during the process. After 71 h of fermentation, 28.8 g/L of lactic acid was produced with a yield and productivity of 0.60 g/g (on total sugars) and 0.51 g/L/h, respectively. No other by-products were detected in the medium.

Sugar bread and crust bread are two starch-rich substrates. Strain A107 was selected for the fermentation using sugar bread and strain A59 for the case of crust bread. After hydrolysis, the sugar bread contained 78.5 g/L of glucose and 60.1 g/L of disaccharides, which is probably a mixture of sucrose and maltose but due to analytic restrictions, it was not possible to separate them. Sugar consumption with parallel lactic acid production started in the first 2 h of fermentation, with a clear preference for glucose. Within 48 h, 84.3 g/L of lactic acid was produced, with a yield of 0.70 g/g and 1.76 g/L/h productivity (Figure 2C). The process was stopped after 64 h since sugar consumption almost ceased.

Strain A59 was selected for the fermentation of crust bread due to its ability to produce its own amylases. The initial sugar concentration was 46.5 g/L, of which, 6.7 g/L was glucose, 3.9 g/L fructose and 35.9 g/L disaccharide (most possibly maltose) (Figure 2D). All sugars were consumed within 16 h with the production of 55.4 g/L of lactic acid. Glucose was not detected again in the medium but the presence of the disaccharide indicated that the strain was able to hydrolyze the residual starch. The process was stopped after 65 h since lactic acid production stabilized at 86.4 g/L. Productivity (at 52 h) was 1.66 g/L/h. Due to the hydrolyzing ability of the strain, it was not possible to calculate the yield of lactic acid from the sugar content.

The fermentation results on the pilot scale demonstrate that lactic acid can be biotechnologically produced from different raw materials. The defined medium resulted in the highest values of final lactic acid concentration (99.5 g/L), yield (0.87 g/g) and productivity (2.11 g/L/h) but the starch based media (crust and sugar bread) were also suitable substrates. There are not many studies in the literature dealing with the production of lactic acid in pilot scales. Karp [14] studied the fermentation of vinasse enriched with soybean molasses for lactic acid production using the strain *Lactobacillus agilis* LPB 56, in an 80 L pilot scale bioreactor. The yield and productivity presented were 0.849 g/g and 0.863 g/L/h, respectively. Coupling the fermentation with a ceramic membrane system for in situ product separation led to an increased lactic acid production both in terms of final concentration and productivity. In the study of Lu [15], the authors reported L(+)-lactic acid concentration of 157.22 g/L and a volumetric productivity of 8.77 g/L/h. Pleissner [7] tested the strain *B. coagulans* for the fermentation of coffee pulp on pilot scales, achieving a yield of 0.78 g/g and a productivity of 4.02 g/L/h. It is obvious that substrate, strain selection, and fermentation mode can highly affect the upstream process.

After the efficient production of lactic acid, its separation and purification from the fermentation broth are equally crucial for polymer grade lactic acid. At the same time, the downstream process should be equally sustainable and environmentally benign. To this end, membrane filtration was applied in all substrates, aiming to separate lactic acid from the other fermentation components.

### 3.2. Microfiltration

Microfiltration is an efficient method for the removal of cell biomass, without major lactic acid losses. The composition of the initial stream (after inactivation), that was fed to the microfiltration, is presented in Table 2.

Table 3 presents the initial volume of every stream, the time required for the microfiltration, the volume reduction (VR), the average permeate flux (J_aver_) as well as the percentage of sugar rejection and lactic acid losses. Fermentation broths derived from crust bread and sugar bread had almost the same final volume and very similar nature since there were both starch-rich substrates. However, microfiltration of the crust bread fermentation broth required much more time in comparison to sugar bread. Moreover, the average permeate flux was approximately 62.3% higher in the sugar bread than in the crust bread. Microfiltration of acid whey was the fastest but the overall volume of the broth was less. As expected, the defined medium was the easiest substrate to microfilter, since, for the initial volume used, the process was faster and presented the highest average permeate flux (263.3 L/m^2^/h).

Among the alternative substrates, the highest losses of lactic acid (21.5%) were observed from the crust bread hydrolysate, whereas from sugar bread the losses were slightly lower (17.5%) (Table 3). No sugar or lactic acid losses were detected when acid whey was microfiltered, whilst from the defined glucose solution, lactic acid losses were only 3.2% (Table 3). Residual sugars were also partially rejected after this processing step and their retention had the same profile as lactic acid; 21.6% from crust bread, followed by 15.4% from sugar bread and 1.6% from the defined glucose solution (Table 3).

**Figure 3:**
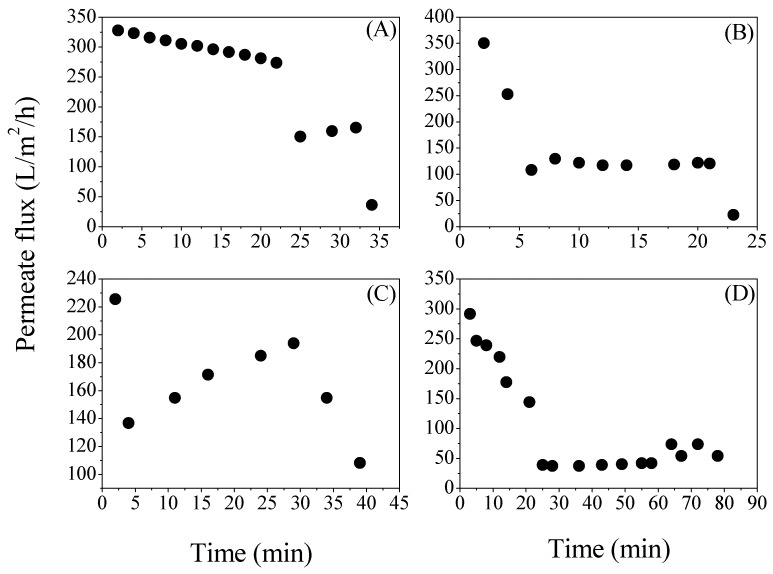
Evolution of the permeate flux (J) during microfiltration of (**A**) defined medium; (**B**) acid whey; (**C**) sugar bread and (**D**) crust bread.

In Figure 3, the evolution of permeate flux is demonstrated. The reduction of the permeate flux is evident for all the studied media. The highest stability was observed when the defined solution was used (Figure 3A). Microfiltration started with a permeate flux of 386.5 L/h/m^2^, which remained approximately at 300 L/h/m^2^ during the process. After about 42 min of filtration, the permeate flux decreased to 150 L/h/m^2^, to end up at 36 L/h/m^2^, due to a pump failure. For the case of acid whey, the permeate flux rate fell abruptly during the first minutes of filtration (from 350.4 L/h/m^2^ to 108.3 L/h/m^2^ after 10 min) but then it remained almost stable (around 117 L/h/m^2^) until the end of the process (Figure 3B). A reduction of the permeate flux during the first 10 min of microfiltration also occurred during the processing of the sugar bread fermentation broth (from 225.6 L/h/m^2^ to 136.8 L/h/m^2^) (Figure 3C). The drop of permeate flux during microfiltration could be attributed to the concentration polarization and the formation of a cake layer from the microbial cells [16,17]. Interestingly, the flux slightly increased over the process (highest value of 194 L/h/m^2^ after 48 min) and decreased again at the end of the filtration (108.3 L/h/m^2^). This increment of the flux could be associated with the rise of the substrate’s temperature. Microfiltration of crust bread started with a constant reduction of the permeate flux until it stabilized at the lowest value of about 40 L/h/m^2^ in comparison to all the other substrates (Figure 3D). At the end of the filtration, the permeate flux was only 15 L/h/m^2^. It is, therefore, quite evident that the nature of the substrate can significantly alter the filtration efficiency and time.

### 3.3. Nanofiltration

#### 3.3.1. Permeate Flux of the Different Substrates

**Figure 4:**
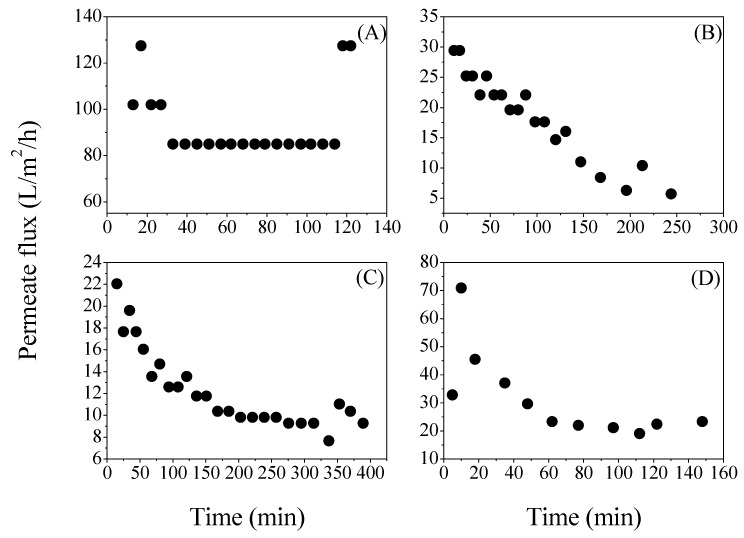
Evolution of the permeate flux (J) during nanofiltration of (**A**) defined medium; (**B**) acid whey; (**C**) sugar bread and (**D**) crust bread.

Nanofiltration was equally affected by the type of substrate employed as in the case of the microfiltration. The J_aver_ was higher for the defined glucose solution (95.9 L/h/m^2^), followed by the crust bread (31.6 L/h/m^2^), acid whey (18.5 L/h/m^2^) and the lowest one was found for the sugar bread substrate (12.4 L/h/m^2^). The profile of the permeate flux during filtration time was also quite different and highly dependent on the substrate (Figure 4).

The permeate flux during the nanofiltration of the defined glucose medium was quite stable and it remained—as in case of microfiltration—for almost the entire process at a value of about 85 L/h/m^2^ (Figure 4A). The addition of 10 L of water close to the end of the filtration resulted in an increase of the permeate flux to its initial values (127.5 L/h/m^2^). Nanofiltration of 103.5 L lasted for 122 min. For sugar bread, a decrease of the permeate flux was observed throughout the filtration (Figure 4C). Initially, the permeate flux was at 22.1 L/h/m^2^ and it slowly decreased to 10 L/h/m^2^ and stabilized until the end of the process. 

When the crust bread hydrolysate was nanofiltered, during the first 5 min, the permeate flux was only 32 L/h/m^2^ but then increased to 70 L/h/m^2^ and stabilized at approximately 20 L/h/m^2^. For a volume of 111 L, 148 min of nanofiltration was required (Figure 4D). The acid whey nanofiltration followed a similar trend to the sugar bread since the decrease of permeate flux was evident (Figure 4B). The initial permeate flux was 29.4 L/h/m^2^ and at the end of the process, it presented a value of 5.7 L/h/m^2^ (244 min). The decline of the permeate flux in all cases was a very common phenomenon in nanofiltration and it is reported to be also correlated with the high lactic acid concentrations [17].

The permeate fluxes during both MF and NF were higher for the defined medium; a result that can be easily attributed to the viscosity and complexity of the waste streams used for fermentation [18]. The highest J_aver_ during NF was achieved using the defined medium or crust bread, with values of 95.9 and 31.6 L/h/m^2^, respectively, whilst NF of the acid whey presented a J_aver_ of 18.5 L/h/m^2^ and of sugar bread as low as 12.4 L/h/m^2^. According to Bouchoux [19], permeate fluxes close to 35 L/h/m^2^ are considered suitable for industrial applications. The authors also studied the NF of two different industrial effluents containing lactic acid using a DK nanofiltration membrane. Kang [20] utilized the NF45 membrane for lactic acid separation from a fermentation broth and the permeate flux reported was 20 L/h/m^2^ at 27 bar. Both DK and NF45 present similar properties to OS-15 used in this study.

#### 3.3.2. Rejection of Sugars, Lactic Acid and Metal Ions

The rejection of lactic acid and the other fermentation impurities is crucial for an efficient separation process since the goal is to produce polymer-grade lactic acid. A suitable membrane will mainly reject the residual sugars and proteins but not lactic acid. The type of substrate and the molecular weight (MW) of the compounds could also highly affect their rejection of the membrane.

The MF permeate of the defined glucose solution contained a very small amount of residual glucose (1.2 g/L), 87.5 g/L lactic acid, as well as residual proteins (expressed as total Kjeldahl nitrogen), phosphorus, and metal anions with Na^+^ being the predominant one, since NaOH was utilized for pH adjustment. The rejection of every component is presented in Table 4. Glucose and lactic acid rejection was 18.5% and 12.5% respectively. The highest rejections were observed for phosphorus with a value of 89.5%, followed by Mg^2+^ (82.8%) and Ca^2+^ (64.5%).

Acid whey contained 13.1 g/L unconsumed sugars, mainly lactose (8.9 g/L) and lower amounts of galactose (4.2 g/L), whilst the lactic acid concentration in the medium was 33 g/L. As expected, lactose rejection was 82%, while galactose rejection was similar to the one of glucose (16.7%), leading to a total sugar rejection of 61.1%. The corresponding value for lactic acid was approximately 10%. As in the case of the defined glucose medium, rejections of phosphorus (55.8%), Mg^2+^ (75.4%) and Ca^2+^ (68.8%) were high.

Sugar rejection for crust bread reached apparently 100% since no sugars were detected in the NF permeate stream. Lactic acid rejection was higher in comparison to the other substrates with a value of 22.4%. Rejections of more than 90% were achieved for phosphorus (98.8%) and for Mg^2+^ and Ca^2+^ (98.9% and 96.6%, respectively).

Residual sugars from sugar bread were 22.7 g/L and they were composed of glucose (9.3 g/L), sucrose (12.3 g/L) and fructose (1.1 g/L). Sucrose rejection was 89.4%, similar to galactose (82.5%), whereas for glucose the rejection was 23.6%, while no fructose was detected in the permeate stream. Lactic acid rejection was the lowest for all of the studied cases with a value of only 2.5%. Rejections of phosphorus, Mg^2+^ and Ca^2+^ were at comparable rates to the previously studied substrates (67.1%, 75.2% and 61.7%).

The sugar rejection from the membrane was directly correlated to the concentrations in the fermentation broth. Goulas [21] studied the effect of different pressures during nanofiltration of a model sugar solution at 25 °C. Their experiments showed that increasing the pressure to 30 bar led to higher sugar rejections and to an increased permeate flux. Sugars are neutral molecules, which means that they are transferred through the NF membranes via convection and diffusion and their rejection is a result of size exclusion [18,22]. According to the study of Pontalier [23], the concentration of sugars in the feed affects their diffusive transport, regardless of the pressure employed. The variations of sugar rejections between the different fermentation broths can be attributed to the different size (monosaccharides versus disaccharides), their concentration in the medium, as well as the nature of other solutes since they can affect the properties of the membrane [18]. As a result, the disaccharides (maltose, sucrose, lactose) were rejected at high rates due to their higher molecular weight (MW = 342.3 g/mol).

Lactic acid rejection is highly affected by the feed composition. The lowest lactic acid rejection (2.5%) was observed for sugar bread, whereas the highest rejection was monitored for crust bread (22.4%). Even though the composition of the two substrates was quite similar, crust bread hydrolysate contained residual starch (approximately 30 g/L), which led to cake formation on the membrane. Interestingly, the lactic acid rejection in the defined medium was equal to 12.5%, which could be mainly attributed to its high initial concentration (87.5 g/L). In any case, lactic acid losses were relatively low and the retentate could be recycled in order to exploit the residual nutrients and recover the remaining lactic acid [8]. These values are in accordance with the cited publications. Bouchoux [19] also reported lactic acid rejections equal to 15 and 18%. Since the pH was around 6.5 for all the fermentation broths, lactic acid was found mainly in its dissociated form, meaning that its rejection from the membrane was due to size exclusion and electrostatic interactions [19]. Li [24] studied the lactic acid recovery from acid whey fermentation using the nanofiltration membranes DS-5DK and DS-5HL. After setting the pH of the fermentation broth at 3.3, lactose retention was more than 90% for the case of DS-5DK but lactic acid recovery was only 76.9% and 50% for DS-5HL. The authors also found that lactic acid concentration in the broth greatly affects lactose retention from the membranes. Sikder [25] tested the NF3 membrane for the purification of lactic acid produced after fermentation of sugar cane juice. The authors reported the retention of 94% of unconsumed sugars but only 32% of lactic acid was recovered in the permeate stream; with a flux of 113 L/m^2^/h.

Besides sugar rejection, the separation of high molecular weight compounds (e.g., proteins) and metal ions is also crucial, since these compounds could decrease the purity of the final product. All of these compounds were present in different concentrations and they had a different molecular size and charge, meaning that the separation mechanisms involved were also different and quite complex. Protein rejection, expressed as total Kjeldahl nitrogen, was significant in all the studied cases. Acid whey contained the highest initial nitrogen content (2368 mg/L) and rejection in the permeate stream was equal to 40%. The highest rejection of total nitrogen was achieved with crust bread (77.5%), followed by sugar bread (59.7%). Efficient separation of phosphorus was also carried out as rejections were more than 50% for all the substrates (Table 4), reaching 89.5% and 98.8% for the defined medium and crust bread respectively. The rejection of ions was equally high (Table 4). Sulphate and magnesium ions presented rejections higher than 70% for all the substrates. In such complex media, the main mechanism of rejection should be a size effect but electrostatic effects should play a role especially for the rejection of sulphate ions [19]. On top of that, sulphate ions have the same size as lactate, so rejections that high should definitely be correlated to charge repulsion effects [26]. Calcium rejections were more than 60% for the defined medium, acid whey, and sugar bread, whereas for crust bread it reached a value of 96.6% (Table 4). Calcium and magnesium rejections of 70 and 60% were previously reported in the literature [19]. Size exclusion should be again the main phenomena involved, even though it has been demonstrated that divalent ions can also interact with the surface on NF membranes, forming complexes [27]. High rejections of sulphate ions were also demonstrated in the study of Mänttäri [28], using the membrane Desal-5 DK for the purification of organic acids via nanofiltration from black liquor. Finally, chloride rejections were the lowest among the studied ions (Table 4). Chloride ions have a lower size and molecular weight to e.g., lactate and phosphate ions, and they could be transferred through the membrane more freely [19].

Table 5 presents the concentrations of lactic acid and the residual impurities of the permeate stream. Nanofiltration was highly efficient in removing residual proteins, phosphorus and metal ions, leading to an increase in the lactic acid purity from 3 to 13%, depending on the substrate. The utilization of commercial nutrient sources led to the highest product purity (77.6%). The starch-based substrates resulted in similar lactic acid purities to the defined medium, after nanofiltration. Acid whey was the least promising substrate since neither the final lactic acid fermentation yield (0.6 g/g) nor the purity after filtration (44.2%) were as high as the other studied media. The nanofiltered media could be further subjected to e.g., bipolar electrodialysis membranes and ion exchange chromatography for achieving polymer-grade purity values.

## 4. Conclusions

A defined medium with glucose, acid whey, sugar bread and crust bread hydrolysates were utilized as fermentation substrates for lactic acid production in pilot scales. The fermented media were then subjected to both microfiltration and nanofiltration in order to separate lactic acid from the majority of the other fermentation components. Microfiltration could efficiently separate the cell biomass and high molecular weight particles without major losses in lactic acid. High rejections of sugars, proteins and metal ions were achieved after nanofiltration, leading to a more than 10% improvement of lactic acid’s purity in the alternative substrates. These results indicate that filtration could be industrially employed as the primary separation step of lactic acid even from complex fermentation broths.

## Figures and Tables

**Table 1:** Material balances (L) of the different substrates after each treatment.

			Microfiltration			Nanofiltration			
Substrate	End of Fermentation	After Inactivation	Permeate	Retentate	Losses	Volume Processed	Permeate	Retentate	Water Addition
**defined medium**	878	941	910	31	0	103.5	105	8.1	10
**acid whey**	320	349	327	22	0	296.8	293.5	33	30
**sugar bread**	735	788	705	67	16	705	718.6	53	72
**crust bread**	730	784	640	119	25	640	637.5	60.9	60

**Table 2:** Composition of the fermentation broths after medium inactivation.

Substrate	Glucose (g/L)	Disaccharide (g/L)	Fru/Xyl/Gal (g/L)	Lactic Acid (g/L)	Monovalent Ions (mg/L)	Divalent Ions (mg/L)
Defined medium	1.26	n.d.	n.d.	90.4	27,083.6	201.0
Acid whey	n.d.	8.9	4.2	33.0	36,657.0	1839.0
Sugar Bread	10.0	13.0	n.d.	77.0	21,207.4	264.4
Crust Bread	n.d.	10.0	n.d.	76.0	20,151.0	402.0

**Table 3:** Initial volume (V_in_), microfiltration time, average permeate flux (J_aver_), volume reduction (VR), sugar rejection and lactic acid losses during microfiltration of the different substrates.

Substrate	V_in_ (L)	Time (min)	VR (%)	J_aver_ (L/m^2^/h)	Sugar Rejection (%)	Lactic Acid Losses (%)*
Defined medium	941	34	3.3	263.3	1.6	3.2
Acid whey	349	23	11.2	143.7	0	0
Sugar bread	788	39	10.5	166.4	15.4	17.5
Crust bread	784	83	18.4	103.8	21.6	21.5

* Lactic acid losses were calculated from the grams of lactic acid in the feed and permeate stream.

**Table 4:** Rejections (R%) of lactic acid, sugars and other nutrients present in the studied substrates after nanofiltration.

Substrate	Total Sugars	Glucose	Disaccharide	Fructose/Galactose	Lactic Acid	Total N	Total P	Cl^−^	SO_4_^2−^	Na^+^	K^+^	Mg^2+^	Ca^2+^
**Defined medium**	18.5	18.5	-	-	12.5	51.0	89.5	25.6	84	24.0	21.7	82.8	64.5
**Acid whey**	61.1	-	82.0	16.7	10.0	39.9	55.8	1.8	88.4	13.5	14.2	75.4	68.8
**Sugar bread**	63.0	23.6	89.4	100.0	2.5	59.7	67.1	6.1	71.4	24.9	25.2	75.2	61.7
**Crust bread**	100.0	-	100.0	-	22.4	77.5	98.8	1.2	68.8	16.6	26.5	98.9	96.6

**Table 5:** Final concentrations (g/L) of lactic acid and fermentation impurities after nanofiltration and lactic acid purity (%), before and after nanofiltration.

Substrate	Total Sugars	Lactic Acid	Total N	Total P	Cl^−^	SO_4_^2−^	Na^+^	K^+^	Mg^2+^	Ca^2+^	LA Purity before NF	LA Purity after NF
**Defined medium**	1.0	76.6	0.4	0.1	0.03	0.02	20.2	0.3	0.004	0.02	74.9	77.6
**Acid whey**	5.1	29.7	1.4	3.7	9.3	0.1	14.0	7.0	0.1	0.05	39.0	44.2
**Sugar bread**	8.4	69.2	0.4	0.7	0.4	0.1	18.2	0.4	0.03	0.09	62.2	70.7
**Crust bread**	0	52.2	0.3	0.01	1.4	0.03	14.5	0.3	traces	traces	68.7	75.9
